# Anorexia nervosa during COVID-19: loss of personal control and alexithymia as important contributors to symptomatology in adolescent girls

**DOI:** 10.1186/s40337-023-00905-w

**Published:** 2023-10-12

**Authors:** Irina Jarvers, Angelika Ecker, Daniel Schleicher, Stephanie Kandsperger, Alexandra Otto, Romuald Brunner

**Affiliations:** Department of Child and Adolescent Psychiatry and Psychotherapy, University of Regensburg, Universitaetsstraße 84, 93053 Regensburg, Germany

**Keywords:** Adolescence, COVID-19, Alexithymia, Loss of control, Social media, Anorexia nervosa

## Abstract

**Background:**

In the course of the COVID-19 pandemic, a steady increase in adolescent anorexia nervosa admissions has been observed. Contributing factors may have been uncontrollable changes in school attendance due to lockdowns and social restrictions. However, patients’ reports on the impact of these factors have not been assessed in detail as of yet. Furthermore, alexithymia, the difficulty to identify and describe one’s own emotions, has increased during the pandemic and is known to be heightened in eating disorders. Thus, it may have contributed to symptom severity in anorexia nervosa during the pandemic.

**Methods:**

The present study examined pandemic-related changes in social media use, body satisfaction, and perceived loss of control and their impact on depressive, anxious, and eating disorder symptomatology in a sample of adolescent girls with anorexia nervosa (*n* = 29) and healthy controls (*n* = 23). Additionally, the influence of current alexithymia as a cross-diagnostic risk factor was assessed. Adolescents answered questionnaires once shortly after admission to inpatient, outpatient, or daycare treatment.

**Results:**

An increase in perceived loss of control during the pandemic and heightened alexithymia explained a significant portion of variance in present depressive symptomatology, which in turn contributed to eating disorder symptomatology.

**Conclusions:**

These relationships emphasize alexithymia and perceived loss of control as valuable constructs for early screenings and interventions.

## Introduction

Anorexia nervosa (AN) is an eating disorder characterized by low body weight, impaired self-perception of shape and weight, and the highest mortality risk of all psychiatric disorders [1]. Perceived loss of control [2] and increased comparisons with others on social media [3] have been postulated as important factors in the etiology of anorexia nervosa. Additionally, recent work has emphasized heightened alexithymia, the difficulty to identify and describe one’s own emotions, in people with eating disorders [4] and argued for its relevance in the emergence of eating disorder symptomatology [5] and negative therapy outcomes [6]. During the COVID-19 pandemic, a worsening of eating disorder symptomatology [7] and a steady increase in admissions of young people with AN was observed [8]. It has been hypothesized that these trends were caused by increased loss of control due to social restrictions and lockdowns [9] as well as increased social media use [10]. Additionally, adolescents’ alexithymia increased during governmental lockdowns [11], which was associated with increases in emotional eating in adults [12]. To our knowledge, no study has specifically surveyed adults or adolescents with AN about pandemic-related changes in perceived personal and environmental control and social media use, and assessed the contribution of alexithymia.

Therefore, the purpose of this study was to examine the impact of changes in body satisfaction, social media use, and loss of control during the COVID-19 pandemic on anxiety, depression, and eating disorder symptomatology in a clinical sample of adolescent girls with AN in comparison to a healthy control group. Additionally, the impact of alexithymia as a general risk factor for AN was examined as a predictor for symptomatology.

## Method

All procedures involving human participants were approved by the Ethics Committee of the University of Regensburg (reference number: 21-2438-101) and the study is registered in the German Clinical Trials Register (DRKS00025963).

### Participants

Adolescent girls with AN were recruited via inpatient, outpatient, and daycare units of the Clinic for Child and Adolescent Psychiatry, Psychosomatics, and Psychotherapy at the University of Regensburg, Germany. Therapists at the clinic asked parents and their daughters whether a member of the study team was allowed to contact them regarding an ongoing scientific study. After permission was granted, a member of the study team contacted the family and explained the purpose of the study as well as inclusion and exclusion criteria. Inclusion criteria were female sex, age between 11 and 19 years, and an official diagnosis of anorexia nervosa, either restrictive, active or atypical (F50.00, 50.01, 50.1). Exclusion criteria were the presence of comorbid autism, neurological disorders, substance addiction or current pregnancy. Healthy controls were recruited via the distribution of study advertisements among employees of the clinic and study posters at local schools and places commonly visited by adolescents like the cinema and youth centres. After choosing to participate, healthy controls and their parents contacted the study team via email or telephone and inclusion and exclusion criteria were checked. Exclusion criteria for controls additionally included the presence of any type of psychiatric disorder or prior inpatient or outpatient psychiatric care. If families agreed to participate and fulfilled all inclusion criteria but no exclusion criteria, they were invited for a study appointment. Overall, *n* = 29 adolescent girls with AN and *n* = 23 adolescent girls without mental health problems participated. The first participant was included on the 14th of December, 2021.

### Procedure

After arriving at the Clinic for Child and Adolescent Psychiatry, Psychosomatics, and Psychotherapy, the participant and their parents were introduced to study objectives and the structure of the appointment. After signing a form to confirm they received sufficient information about the study, adolescents and their guardians provided their written informed consent and participated in an appointment for psychiatric evaluation which consisted of several questionnaires and a clinical interview. The assessment was part of a larger longitudinal study examining possible influences on treatment outcome.

During the assessment, participants filled out questionnaires regarding anxiety, depression, eating disorder severity, and alexithymia. The appointment lasted approximately 2 to 2,5 h.

### Measures

#### Depressive and anxious symptomatology

Beck’s Anxiety Inventory (BAI) [13] was used for anxiety symptoms and Beck’s Depression Inventory-2 (BDI-II) [14] for depression symptoms. Both self-report questionnaires assess cognitive, emotional and behavioural symptoms of anxiety and depression. The BAI consist of 21 items that are rated on a scale from 0 (not at all) to 3 (strongly). Psychometric properties of the BAI are good with an internal consistency of Cronbach’s α = 0.90 and a test–retest reliability of r = 0.73 [13]. The BDI-II also consists of 21 items with an identical 4-point scale and is frequently used in adolescents. Good internal consistency between > 0.90 and test–retest reliability of r > 0.80 has been identified [15].

#### Eating disorder symptomatology

The Eating Disorder Inventory-2 (EDI-II) [16] is a self-report questionnaire that assesses behavioural and psychological eating disorder symptoms and can be used by adolescent girls with AN as well as controls. It contains 91 items which are answered on a 6-point Likert scale and arranged on 11 scales: drive for thinness, bulimia, body dissatisfaction, ineffectiveness, perfectionism, interpersonal distrust, interoceptive awareness, maturity fears, ascetism, impulse regulation and social insecurity. It has shown high internal consistency with values between 0.73 and 0.93 and good test–retest reliability with r = 0.75–0.94) [17, 18]. Good convergent and discriminative validity have also been confirmed [16].

#### Alexithymia

Alexithymia was measured using the German version of the Perth Alexithymia Questionnaire, adapted for children and adolescents (PAQ-C) [19, 20]. The PAQ-C consists of 24 items and three main subscales: difficulties identifying feelings (DIF), difficulties describing feelings (DDF) and an externally oriented thinking style (EOT). Additionally, compound scores can be created with DIF and DDF as well as additional scores depending on whether difficulties occur with positive or negative feelings. Items are scored on a 7-point Likert scale ranging from ‘strongly disagree’ to ‘strongly agree’. For the English, Polish, and Spanish version excellent validity and reliability have been reported in addition to internal consistencies of α = 0.90–0.96 [20–22]. In the present sample the internal consistency was α = 0.96.

#### Psychiatric assessment

The Mini-International Neuropsychiatric Interview for Children and Adolescents (MINI-KID 6.0) [23] was used to confirm diagnoses in adolescent girls with AN and screen healthy controls for psychiatric disorders. The MINI-KID covers a broad spectrum of diagnoses in children and adolescents and shows excellent inter-rater reliability (> 0.89 – > 0.94), test–retest reliability (0.87 – 1.00) as well as validity by correlating with other assessments of psychopathology [24]. In Table 1 official diagnoses from the diagnostic procedure at the clinic are reported.

**Table 1:** Descriptive statistics including diagnoses and group differences

Variables	AN Group	Control Group	Group comparisons
Sample size	29	23	
Age			*Z* = − 0.39, *p* = 0.694
M	14.90	15.13	
SD	1.61	1.55	
Range	12–18	12–18	
BMI in kg/m^2^			*Z* = − 5.18, *p* < 0.001
M	15.68	20.25	
SD	0.98	2.90	
BAI			*Z* = − 3.65, *p* < 0.001
M	20.90	7.43	
SD	13.54	5.65	
BDI-II			*Z* = − 5.31, *p* < 0.001
M	26.93	5.43	
SD	14.70	4.97	
EDI-II			*Z* = − 4.50, *p* < 0.001
M	302.76	200.35	
SD	82.21	36.66	
PAQ			*Z* = − 2.01, *p* = 0.045
PAQ Total Score			
M	80.34	60.17	
SD	34.71	25.06	
Diagnoses (ICD-10)	*N*	*N*	
F50.00 Anorexia nervosa, restrictive type	23	–	
F50.01 Anorexia nervosa, active type	5	–	
F50.1 Atypical anorexia nervosa	1	–	
F32.1 Moderate depressive episode	10	–	
F33.1 Recurrent depressive disorder, moderate episode	1	–	
F40.1 Social phobia	2		
F42.2 Mixed obsessional thoughts and acts	2	–	
F66.0 Sexual maturation disorder	1	–	
F81.0 Specific reading disorder	1	–	

*AN* anorexia nervosa, *BAI* Beck’s Anxiety Inventory, *BDI-II* Beck’s Depression Inventory-2, *EDI-II* Eating Disorder Inventory-2, *PAQ* Perth Alexithymia Questionnaire, *DAF* Difficulties Appraising Feelings, *ICD-10* International Statistical Classification of Diseases and Related Health Problems, 10th Revision. For the EDI-II a total score summed over all scales is reported. Several diagnoses were possible per person

#### COVID-19 specific changes

To measure changes in family relationships, body satisfaction, media use, and perceived loss of control, the COVID-19 Anorexia Questionnaire (COV-AN )[25] was developed by our department. The COV-AN differentiates between environmental and personal control. In the present sample, it showed an internal consistency of α = 0.87. The COV-AN consists of 18 items which are all rated on a scale from -3 ‘much less’ over 0 ‘no change’ to + 3 ‘much more’. Specifically, adolescents were asked to compare their experience before the onset of the pandemic (and Germany-wide lockdowns) to their experience after pandemic onset (e.g., ‘How often since the onset of the pandemic did you feel like everything around you is spinning out of control?’). In Germany, two major lockdowns took place (March–May 2020; December 2020–May 2021) which were both characterized by contact restrictions and shop, restaurant, and school closures. All participants were surveyed after the second lockdown.

### Data analysis

The sample size was determined by the average number of adolescent girls with AN that are admitted within a year. G*Power[26] was used to conduct an a priori power analysis for a linear regression predicting eating disorder symptoms with an alpha of 0.05, power of 0.8, and 7 predictors (social media use, body satisfaction, perceived loss of control, depressive symptoms, anxiety symptoms, alexithymia, and group assignment). An effect size of *f*^2^ = 0.39 was assumed, determined via squared multiple correlations with each predictor correlating at least *r* = 0.20 in order to be included. This criterion was satisfied by all predictors with the smallest correlation being Kendall’s τ = 0.32. The power analysis determined a total sample size of *n* = 42. Group differences on COV-AN items were examined via FDR-corrected Mann–Whitney *U* tests [27], followed by regressions with anxiety (BAI), depression (BDI-II), and eating disorder (EDI-II) scores as dependent variables and body satisfaction, media use, loss of control (COV-AN), alexithymia (PAQ), BAI/BDI-II, and group assignment as independent variables.

## Results

Descriptive statistics and official diagnoses are depicted in Table 1.

### Group comparisons

**Fig. 1:**
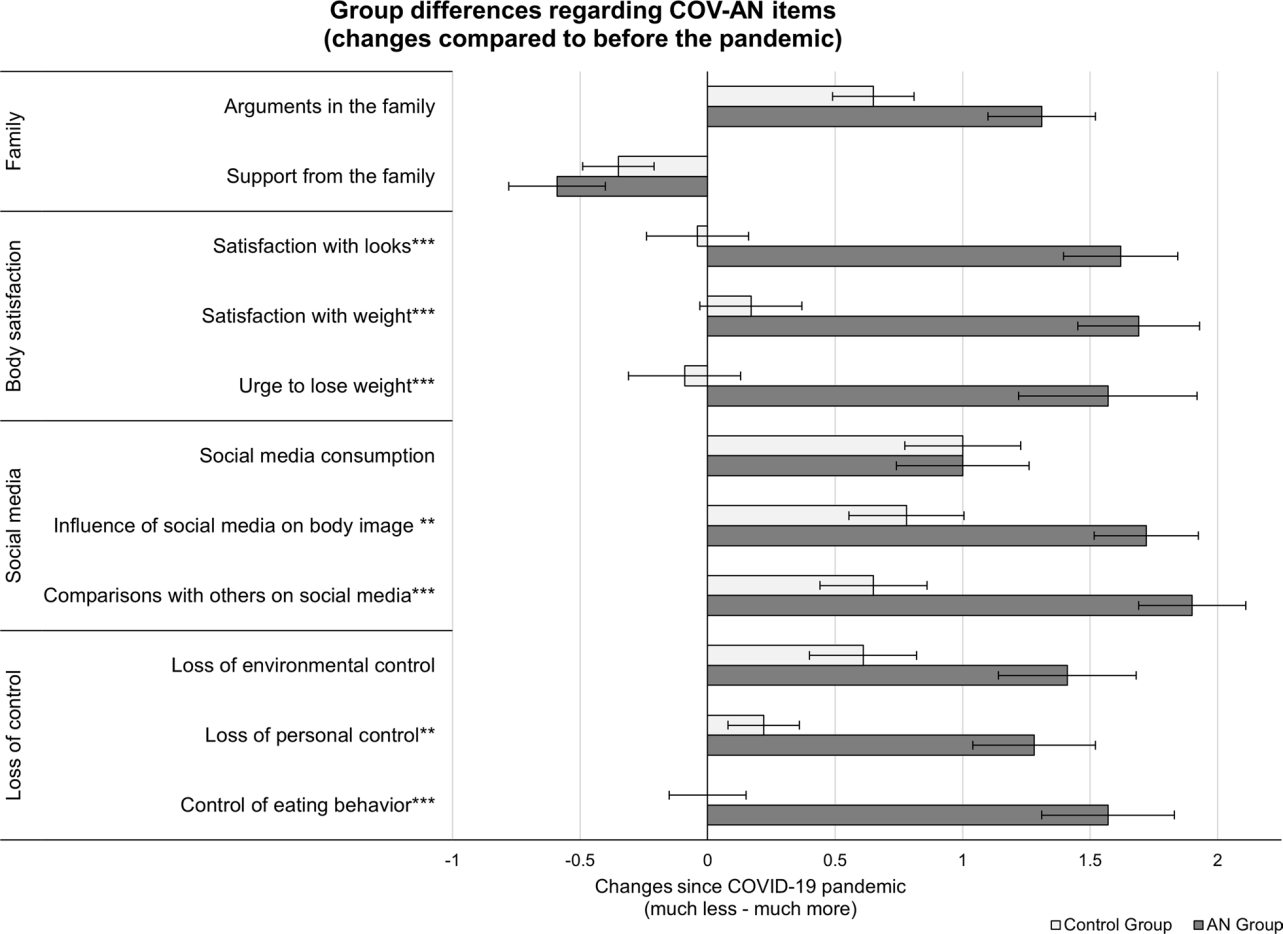
Overview of group comparisons regarding COV-AN items. Differences between the group of girls with anorexia nervosa (AN) and the control group are depicted regarding the COV-AN (COVID-19 Anorexia Nervosa Questionnaire) scales ‘family’, ‘body satisfaction’, ‘social media’ and ‘loss of control’. Group differences on specific items are reported. *p < .05, **p < .01, ***p < .001

Adolescent girls with AN showed more depression, anxiety, eating disorder symptoms and higher alexithymia than controls. Differences in COV-AN items are depicted in Fig. 1. Both groups did not differ in their reports on family dynamics (increase in arguments, decrease in support), on increased social medial consumption, or feeling a loss of environmental control compared to the time before the pandemic. However, adolescent girls with AN reported that they felt more influenced by social media and compared themselves more often with others, as well as an increased urge to lose weight and, as a consequence, higher satisfaction with their weight and body. Furthermore, only girls with AN reported increased loss of personal control and that they used eating restrictions as an attempt to regain that control. When asked whether the pandemic generally influenced their eating disorder, 60.7% of girls with AN reported that it did with 100.0% reporting that the influence was negative.

### Predicting depressive, anxious and eating disorder symptomatology

Linear regression models were computed for anxiety (BAI), depression (BDI-II), and eating disorder (EDI-II) symptoms over both groups (Table 2). Variance in BAI scores could only be explained significantly by BDI-II scores and not by loss of control, media use, body satisfaction, PAQ scores, or group assignment (*F*(6,51) = 16.83, *p* < 0.001). Variance in BDI-II scores could be significantly explained by loss of control, PAQ scores, BAI scores, and group assignment, but not by media use or body satisfaction (*F*(6,51) = 34.16, *p* < 0.001). Finally, variance in EDI-II scores could be explained by BDI scores and PAQ scores, but not by BAI, group assignment, or any COV-AN variables (*F*(7,51) = 49.27, *p* < 0.001).

**Table 2:** Results of linear regression models predicting adolescent girls’ depression, anxiety and eating disorder symptomatology

Dependent Variable	Predictor	*B*	*SE*	*β*	*t*	*p*	*CI B*	*R*^*2*^
Anxiety symptoms (BAI)	COV-AN Loss of Control	0.36	1.61	0.03	0.22	0.825	− 2.89–3.61	0.69
	COV-AN Media Use	0.47	1.45	0.04	0.32	0.749	− 2.45–3.38	
	COV-AN Body Dissatisfaction	1.62	1.61	0.15	1.01	0.318	− 1.61–4.86	
	BDI-II	0.54	0.14	0.66	3.95	< 0.001	0.26–0.81	
	PAQ (Alexithymia)	0.04	0.04	0.09	0.80	0.427	− 0.05–0.12	
	Group	− 2.11	3.19	− 0.08	− 0.66	0.511	− 8.53–4.31	
Depression symptoms (BDI-II)	COV-AN Loss of Control	3.01	1.46	0.20	2.06	0.045	0.06–5.95	0.82
	COV-AN Media Use	0.30	1.37	0.02	0.22	0.830	− 2.47–3.06	
	COV-AN Body Dissatisfaction	0.34	1.54	0.03	0.22	0.828	− 2.76–3.43	
	BAI	0.48	0.12	0.39	3.95	< 0.001	0.24–0.73	
	PAQ (Alexithymia)	0.11	0.04	0.22	2.72	0.009	0.03–0.18	
	Group	8.72	2.74	0.28	3.18	0.003	3.20–14.24	
Eating disorder symptoms (EDI-II)	COV-AN Loss of Control	− 8.52	6.51	− 0.11	− 1.31	0.197	− 21.64–4.60	0.89
	COV-AN Media Use	6.17	5.84	0.07	1.06	0.297	− 5.61–17.94	
	COV-AN Body Dissatisfaction	3.28	6.55	0.05	0.50	0.619	− 9.92–16.48	
	BAI	1.92	0.60	0.29	3.20	0.003	0.71–3.14	
	BDI-II	2.79	0.64	0.53	4.40	< 0.001	1.51–4.07	
	PAQ (Alexithymia)	0.48	0.18	0.18	2.68	0.010	0.12–0.84	
	Group	6.89	12.92	0.04	0.53	0.597	− 19.15–32.92	

*BAI* Beck’s Anxiety Inventory, *BDI-II* Beck’s Depression Inventory-2, *EDI-II* Eating Disorder Inventory-2, *PAQ* Perth Alexithymia Questionnaire, *COV-AN* COVID-19 Anorexia Questionnaire

## Discussion

Overall, while both healthy adolescent girls and adolescent girls with AN were negatively impacted by the pandemic, especially girls with AN reported suffering from loss of personal control and subsequent food restrictions in addition to more comparisons with others on social media. Perceived loss of control, alexithymia, and anxiety symptoms could explain a significant portion of variance in depression symptoms, which in turn predicted eating disorder symptomatology. Prior qualitative work mentions loss of control as a possible initiator of eating disorder symptoms [2], arguing that uncontrollable events contribute to a feeling of lost control, which is then compensated by restricting (controlling) food intake. As the COVID-19 pandemic had a sudden onset and quarantines were government controlled, it may have served as a trigger event for the adolescent girls with AN in the present study. Social media use has also been associated with increased eating disorder risk, in particular when time was spent on appearance-related behaviours [3]. This association could not be confirmed in the present study. It is possible that comparing oneself to others on social media is a consequence of appearance-related behaviours and not vice versa. A longitudinal study examining cross-lagged relations between social media use and symptom development would be able to address this question in more detail. Finally, alexithymia was an important risk factor for both, depressive and eating disorder symptomatology. Longitudinal work by Li et al. [28] showed that alexithymia served as a mediator between perceived stress and mental health problems during the pandemic, emphasizing that alexithymia may have contributed to more overwhelm as a consequence of restrictions and loss of control.

A limitation of the present study is that the questionnaires were answered retrospectively, both by adolescent girls with AN and controls. This was difficult to circumvent, since girls with AN searched for help after symptom onset. Strengths are a detailed characterization of adolescent girls with AN, psychiatric screenings of controls, and the assessment of specific domains of daily life impairments during the pandemic. In the future, it might be of great value to screen for alexithymia and to provide alternative methods of regaining personal control, besides food restrictions, to aid adolescent girls with anorexia nervosa, especially in situations that involve increased loss of control like a global pandemic[29].

## Data Availability

The datasets used and/or analysed during the current study are available from the corresponding author upon reasonable request.

## Abbreviations

AN: Anorexia nervosa
BAI: Beck’s anxiety inventory
BDI-II: Beck’s depression inventory-2
COV-AN: COVID-19 anorexia nervosa questionnaire
EBI-II: Eating disorder inventory-2
MINI-KID 6.0: Mini-international neuropsychiatric interview for children and adolescents
PAQ-C: Perth alexithymia questionnaire, adapted for children and adolescents
